# Convulsive Stress Mimics Brain Hypoxia and Promotes the P-Glycoprotein (P-gp) and Erythropoietin Receptor Overexpression. Recombinant Human Erythropoietin Effect on P-gp Activity

**DOI:** 10.3389/fnins.2019.00750

**Published:** 2019-07-17

**Authors:** Amalia Merelli, Alberto Javier Ramos, Alberto Lazarowski, Jeronimo Auzmendi

**Affiliations:** ^1^Departamento de Bioquímica Clínica, Instituto de Investigaciones en Fisiopatología y Bioquímica Clínica (INFIBIOC), Facultad de Farmacia y Bioquímica, Universidad de Buenos Aires, Buenos Aires, Argentina; ^2^Laboratorio de Neuropatología Molecular, Instituto de Biología Celular y Neurociencia “Prof. E. De Robertis” IBCN-UBA-CONICET, Universidad de Buenos Aires, Buenos Aires, Argentina

**Keywords:** rHu-EPO, EPO-R, P-gp, hypoxia, epilepsy

## Abstract

Erythropoietin (EPO) is not only a hormone that promotes erythropoiesis but also has a neuroprotective effect on neurons attributed to its known anti-apoptotic action. Previously, our group has demonstrated that recombinant-human EPO (rHu-EPO) can protect neurons and recovery motor activity in a chemical focal brain hypoxia model (Merelli et al., 2011). We and others also have reported that repetitive seizures can mimic a hypoxic- like condition by HIF-1α nuclear translocation and high neuronal expression P-gp. Here, we report that a single 20-min status epilepticus (SE) induces P-gp and EPO-R expression in cortical pyramidal neurons and only P-gp expression in astrocytes. *In vitro*, excitotoxic stress (300 μM glutamate, 5 min), can also induce the expression of EPO-R and P-gp simultaneously with both HIF-1α and NFkB nuclear translocation in primary cortical neurons. Primary astrocytes exposed to chemical hypoxia with CoCl_2_ (0.3 mM, 6 h) increased P-gp expression as well as an increased efflux of Rhodamine 123 (Rho123) that is a P-gp substrate. Tariquidar, a specific 3^er^ generation P-gp-blocker was used as an efflux inhibitor control. Astrocytes treated with rHu-EPO showed a significant recovery of the Rho123 retention in a similar way as seen by Tariquidar, demonstrating for first time that rHu-EPO can inhibit the P-gp-dependent efflux activity. Taking together, these data suggest that stimulation of EPO depending signaling system could not only play a central role in brain cell protection, but this system could be a new tool for reverse the pharmacoresistant phenotype in refractory epilepsy as well as in other pharmacoresistant hypoxic brain diseases expressing P-gp.

## Introduction

Erythropoietin receptor (EPO-R) has been detected in several non-hematopoietic mature cells, usually induced by hypoxic conditions. Irrespective to the erythropoietic stimulation, erythropoietin (EPO) action on EPO-R plays a central role inducing a broad range of cellular responses aimed to preserve the integrity of stressed cells and to protect different cells from hypoxia or other stress signaling (Lappin, 2003). EPO-R has been classically regarded as the EPO main receptor, however, recent data shows that EPO can be active through canonical EPO-R or non-canonical receptors such as Ephrin receptors or transient receptor potential vanilloid (TRPV) channels (see for revision Bond and Rex, 2014; Malik and Di Benedetto, 2018).

The prototypical hypoxia response in eukaryotic cells is the activation of several transcription (HIF-1α factors, particularly the hypoxia-inducible factor 1α), which induces the expression of a wide spectrum of pro-survival genes, including both EPO and EPO-R (Semenza, 2000; Ruscher et al., 2002). In spite of the fact that this robust mechanism is universal for all cell types under similar hypoxic conditions, EPO and EPO-R expression levels are not similar for all cell types from the same tissues (Semenza, 2000). Additionally, HIF-1α stabilization is also detected stimuli even under normoxic condition. For example, the redox-sensitive transcription factor NF-κB (nuclear factor kappa-B) usually involved in inflammation and immune responses (Liu et al., 2017), can also bind to a distinct element in the proximal promoter of the HIF-1α gene (Görlach and Bonello, 2008).

Both transcription factors HIF-1α and NF-κB, can activate MDR-1/ABCB1gene inducing the expression of P-glycoprotein (P-gp). After their discovery in chemotherapeutic resistant cancer (Juliano and Ling, 1976), P-gp was the first ABC-transporter described to be related with drug-resistant epilepsy as well as in brain and heart hypoxia-ischemia (Tishler et al., 1995; Lazarowski et al., 1999, 2004, 2005, 2007; Ramos et al., 2004). In hypoxic conditions, HIF-1α is the master transcriptional regulator of cellular and developmental response to oxygen deprivation in all tissues. Consequently, the stabilization and nuclear translocation of HIF-1α is observed and a wide spectrum of genes are up-regulated, that include the EPO-R and P-gp (Comerford et al., 2002; Aviles-Reyes et al., 2010; Merelli et al., 2011; Semenza, 2014; Auzmendi et al., 2018). Furthermore, seizures generate conditions producing acute brain hypoxia and ischemia followed by inflammation. Some common sequential events including neurotoxicity, depolarization, inflammation and apoptosis have been observed, including the phosphatidylserine exposition on the outer plasmatic membrane (Bateman et al., 2008; Moseley et al., 2010).

A large body of evidence has demonstrated that EPO has several extra-hematopoietic protective effects to a wide spectrum of hypoxic cells and tissues, including cardiovascular, immunity and nervous systems. In Central nervous system (CNS), EPO promotes neuronal development, prevents cell death of damaged neurons, and improves learning and memory (Morishita et al., 1997; Lu et al., 2005; Al-Qahtani et al., 2014; Badowska-Kozakiewicz et al., 2017). Interestingly, several conditions such as hypoxic, traumatic, or inflammatory stresses also can induce P-gp overexpression (Kooij et al., 2012; de Lemos et al., 2013; Jullienne et al., 2016; Willyerd et al., 2016).

In an experimental brain ischemic model previously developed by our group, direct brain intracortical CoCl_2_ injection induced HIF-1α nuclear translocation and high EPO-R and P-gp expression in neurons (Caltana et al., 2009). In this experimental paradigm, intranasal recombinant human EPO (rHu-EPO) administration had not only an effective therapeutic response by recovery of motor activity, but also had a neuroprotective action at cellular level on hypoxic damaged neurons (Merelli et al., 2011). However, the potential effect of EPO on the efflux activity of P-gp in cells of CNS has not been investigated yet.

Since hypoxia and epilepsy shared a partially similar signaling pathways, we hypothesized that excitotoxic stress could induce overexpression of both EPO-R and P-gp. Here, we evaluated *in vivo* and *in vitro* the neuronal and glial induced-expression of P-gp and EPO-R after excitotoxicity. Additionally, we investigated the effect of rHu-EPO over the P-gp activity. Our results support the idea of the excitotocixity could mimic to hypoxia by inducing EPO-R expression thus allowing the therapeutic with rHu-EPO in refractory epilepsy and other pathological stated where P-gp has a major role.

## Materials and Methods

### Ethics Statement

All procedures involving animals and their care were conducted in accordance with our institutional guidelines, which comply with the NIH guidelines for the Care and Use of Laboratory Animals and the principles presented in the Guidelines for the Use of Animals in Neuroscience Research by the Society for Neuroscience, and were approved by the CICUAL committee of the School of Medicine University of Buenos Aires (Res. Nr. 1278/2012). All efforts were made to minimize animal suffering and to reduce the number of animals used.

### Reagents

Cell culture reagents were obtained from Invitrogen Life Technologies (Carlsbad, United States). Fetal calf serum (FCS) was purchased from Natocor (Córdoba, Argentina). Antibodies were purchased from Santa Cruz (rabbit polyclonal anti-p65 cat# SC-8008 and polyclonal rabbit anti-Epo-R), Abcam (mouse polyclonal anti-P-gp, cat# ab3083), and Sigma (polyclonal rabbit antiHIF-1α cat# HPA001275). Poly-L-lysine, DAPI (4′6-diamidino-2-phenylindole), Rhodamine 123 (Rho123), 3rd generation P-gp inhibitor Tariquidar and other chemicals were obtained from Sigma (United States). Fluorescent secondary antibodies were purchased from Jackson Immunoresearch (United States). Microscopic images were taken using an Olympus IX- 81 microscope equipped with a DP71 camera (Olympus, Japan).

### Cell Culture and Treatments. Neuronal Culture

Wistar rat embryos of 17 days (E17) were used for primary cortical neurons culture as previously described (Villarreal et al., 2011). Briefly, embryos were removed from the maternal uteri, brains were surgically removed and cortices were separated under sterility. After a treatment with 0.25% trypsin cortical neurons were mechanically dissociated through a Pasteur pipette. Neurons were placed in a plastic multi-well plate coated with poly-L-lysine. Cells were maintained with NeuroBasal (Invitrogen) supplemented with 2% B27 (Invitrogen) and 0.5 mM glutamine. After 7 days *in vitro* (7DIV), neurons were exposed to excitotoxicity during 5 min with 300 μM glutamate at. Then, neurons were washed and medium was replaced by supplemented NeuroBasal. After 24 h of recovery, neurons were fixed 4% paraformaldehyde plus 4% sucrose in PBS pH 7.2 for 15 min at room temperature.

### Glial Culture

Primary cortical glial cell cultures were obtained from 3 to 5 post-natal day rats as described previously (Rosciszewski et al., 2018). Briefly, brains were separated from the skull and meninges were removed. Cortices were dissected and mechanically disrupted with in Dulbecco’s modified Eagle medium (DMEM). After centrifugation dissociated glial cells were resuspended in DMEM supplemented with 10% FCS, 2 mM L-glutamine, and 100 μg/ml penicillin-streptomycin. Then, glial cells were plated in 96-multi-well plate and were incubated at 37°C and 5% CO_2_ for 7 days until reaching the confluence and medium was replaced every 48 h.

### Status Epilepticus (SE) Protocol

Status epilepticus was induced in 230–250 g Wistar male rats using the lithium -pilocarpine paradigm as previously described (Rossi et al., 2013, 2017). Briefly, sodium lithium chloride was injected (127 mg/kg- i.p), and 20 h later pilocarpine (30 mg/kg, i.p.), were administrated. Severity of seizures were evaluated according to the Racine scale (Racine, 1972) and the rats were considered to have suffered SE when remained more than 5 min with myoclonic and/or tonic-clonic generalized seizures (Racine stages 4 and 5). After 20 min, SE was stopped applying dizepam (20 mg/kg i.p.). All animals were kept under intense care during the initial 24 h to improve animal recovery from seizures and to maintain hydration and feeding. A volume of 0.5 ml of saline was administered every 12 h to maintain hydration. The control non-convulsive group was injected with a lithium-saline solution. After that initial critical 24 h period, animals were routinely monitored until sacrificed at 15 days post-SE (15 DPSE).

### Animal Fixation and Immunohistochemistry

At 15 DPSE, animals were deeply anesthetized with ketamine/xylazine (90/10 mg/kg i.p.), and sacrificed by intracardiac perfusion with 4% paraformaldehyde in phosphate buffer as previously described (Rossi et al., 2013). Frozen brains were cut into 30-μm thick sections and were cryopreserved in a 0.1 M phosphate buffer saline (PBS) containing 20% glycerol and 30% ethylene-glycol. The sections were stored at −20°C until use. For immunohistochemistry, sections were washed with PBS and permeabilized with 1% Triton X-100 in PBS (PBS-X) at room temperature. The unspecific binding was prevented by 1 h incubation with 10% normal goat serum (NGS) in PBS-X at room temperature. Then sections were incubated with primary antibodies diluted in PBS-X plus 3% NGS at 4°C during 48 h. Anti EPO-R and anti-P-gp antibodies were used at 1:400 dilution. After washing, sections were incubated with the appropriate fluorescent secondary antibodies at 1:800 dilution for 4 h at room temperature. Secondary antibodies fluorochromes were Alexa 488 or Alexa 594 (Jackson Immunoresearch). Rat speen sections (10 μm) were used for testing EPO-R immunoreactivity.

### Primary Culture Immunocytochemistry

Neuronal or glial primary cultures were fixed with 4% paraformaldehyde plus 4% sucrose in PBS pH 7.2 for 15 min at room temperature. Only when immunocytochemistry was performed against intracellular epitopes, neurons were permeabilized with PBS-X for 15 min. Unspecific binding was blocked by a 30 min incubation with 5% NGS in PBS at room temperature. Then, primary antibody incubation was performed 24 h at 4°C in a solution containing the respective antibody in 5% NGS in PBS. After 3 washes in PBS, cells were incubated with the appropriate fluorescent secondary antibodies (Alexa 488 or Alexa 594) for 2 h at room temperature. Primary antibodies were used 1:400 dilution while secondary antibodies were used 1:800. Nuclear counterstaining was routinely done with DAPI.

### P-gp Activity Assay

Cortical glial cultures were used to study the effect to rHu-EPO over P-gp activity. Astrocytes were exposed to chemical hypoxia by incubation with 0.3 mM CoCl_2_ in complete DMEM for 6 h at 37°C. Following chemical hypoxia induction, glial cells were washed and then medium was replaced complete serum free DMEM containing rHu-EPO 30–300 IU/ml for 30 min. Then, cells were allowed to load Rho123 by incubating them in serum free DMEM containing 1 μM Rho123 for 5 min. After several washes, we determined the Rho123 retention with a fluorescence microscope. Tariquidar 5 μM was used as a specific blocker of P-gp. rHu-EPO was prepared from a stock solution of 10^6^ IU/ml in dimetilsulfoxide (DMSO).

### Quantification and Statistical Analysis

Morphometrical and densitometric analysis were performed with ImageJ (NIH) and statistical analysis was done with Graph Pad prism software. The area covered by the immunostained cells and the fluorescence intensity were evaluated. After checking the normal distribution of the data, differences were analyzed by one-way ANOVA and Student Newman Keuls post-test comparison or by two tailed Student *t*-test.

## Results

### Convulsive Stress Promotes the P-gp and EPO-R Expression

**FIGURE 1:**
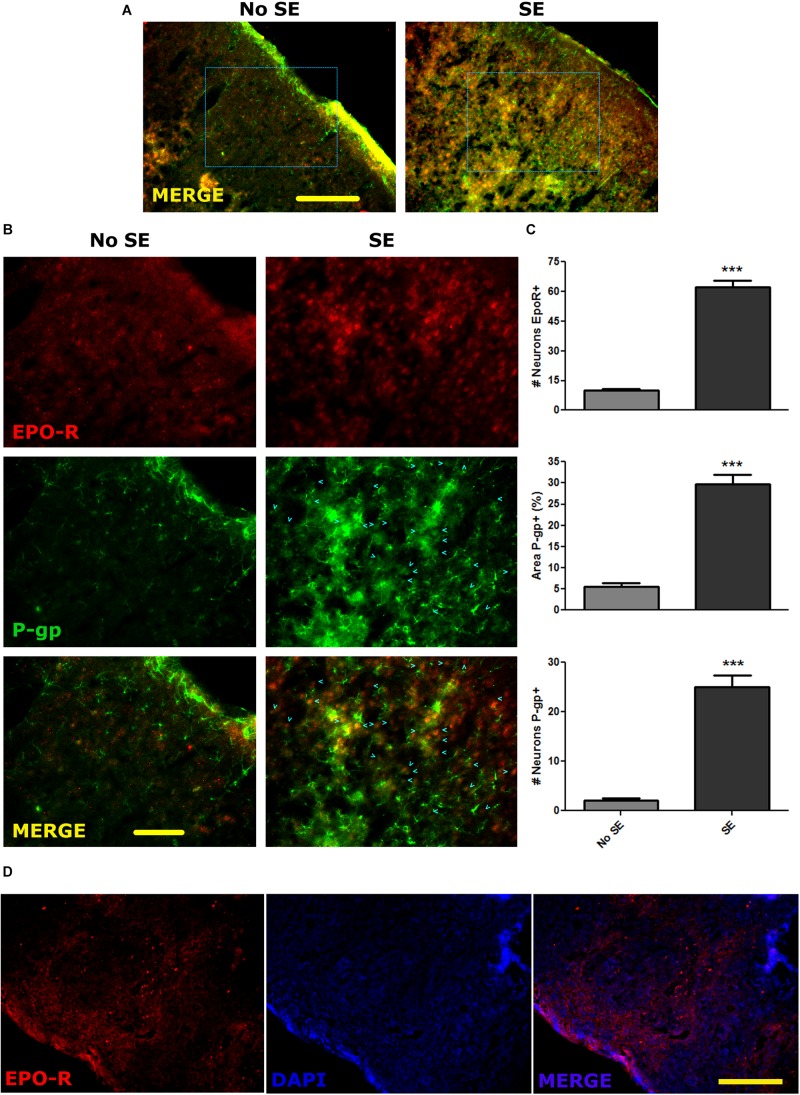
P-gp and EPO-R increased expression after status epilepticus. **(A)** Representative images of EPO-R and P-gp immunostaining in brain cortical sections of rats by 15 days post-SE (DPSE). **(A)** Low magnification images of the entire cortical layers (bar = 0.5 mm). **(B)** Larger magnification of the dashed squares from **A**. (bar = 0.1 mm). Cyan arrow heads represent pyramidal cortical neurons expressing P-gp. **(C)** The graphs show the quantification of the immunostaining showed as the number of neurons per field that express P-pg or EPO-R. Alternatively, the area covered by P-gp staining also shows a significant increase after SE. We analyzed five representative images per animal (three animals per treatment) and differences were analyzed by two tailed Student *t*-test that showed a significance level of *p* < 0.001 (^∗∗∗^). **(D)** Sections of spleen as a positive control of EPO-R antibody binding (bar = 0.5 mm).

Convulsive stress produced by SE induces a myriad of changes in gene expression in neurons and glial cells. These alterations in gene expression and network remodeling are hypothesized to have a main role in the natural story of epilepsy. Because seizures produce an acute hypoxic condition, we first studied if SE was able to induce a long term change in P-gp and EPO-R expression *in vivo*. For that purpose, we induced a single SE and analyzed the P-gp and EPO-R expression by immunohistochemistry at 1 day post SE. As shown in the Figures 1A,B, the P-gp and EPO-R expression was increased in brain cortical glia and neurons after SE. Quantitative analysis of P-gp immunostained area per field, including glial cells and neurons, showed a 5-times increase in P-gp expression (Figure 1C). The number of pyramidal neurons expressing P-gp was increased about 7 times. Additionally, a 4-times increase in the number of pyramidal neurons expressing EPO-R was observed (Figure 1C). EPO-R expression was not clearly found in cells with astrocytes while P-gp expression was present in either astrocytes or neurons. Specificity of EPO-R antibody was tested using spleen sections as positive control (Figure 1D).

### Excitotoxic Stress and P-gp and EPO-R Expression *in vitro*

**FIGURE 2:**
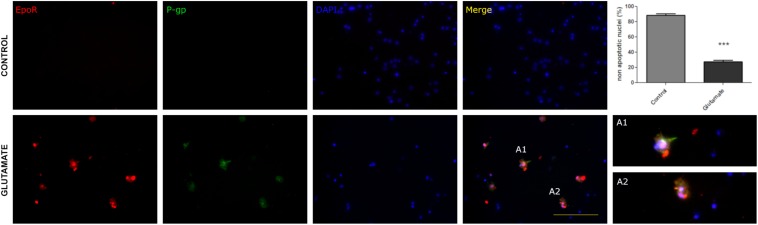
Excitotoxic stress promotes the P-gp and EPO-R expression *in vitro* in primary cortical neurons. Representative images showing the neuronal expression of P- gp (green), EPO-R (red) and nuclei (blue) in control conditions and after exposing neurons to glutamate excitotoxicity by 5 min 300 uM glutamate. Merge show the co-localization while A1 and A2 are expanded view of neurons that co-localized EPO-R and P-gp. Bar = 50 μm. Note that excitotoxicity induced EpoR and P-gp expression in cortical neurons. The graph shows that this excitotoxicity level significantly affects neuronal survival evaluated as nuclear morphologies. Differences were analyzed by two tailed Student’s *t*-test that showed a significance level of *p* < 0.001 (^∗∗∗^).

Since the status epilepticus can produce a wide spectrum of biological responses including hypoxia-ischemic stress, P-gp and EPO-R could be expressed as a consequence of the hypoxia rather than the excitotoxic-convulsive stress itself. To separate the hypoxia from convulsive stress effects we used a primary cortical neurons culture under normoxia. To mimic the convulsive stress, neurons were exposed to a brief pulse of excitotoxic glutamate concentration (0.3 mM). The excitotoxic stimulation significantly reduced neuronal survival evaluated as the abundance of non-apoptotic neurons (Figure 2) but surviving neurons expressed both P-gp and EPO-R (Figure 2).

**FIGURE 3:**
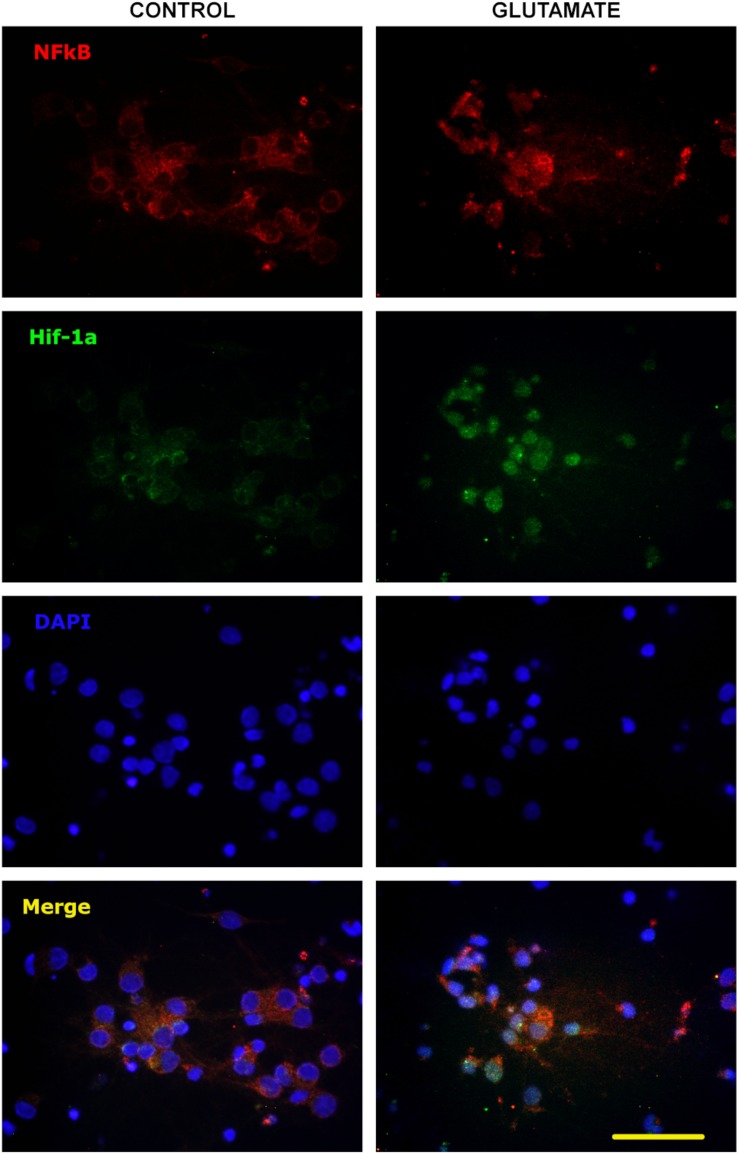
Nuclear translocation of NF-κB and HIF-1α after excitotoxic stress. Representative images of 10 DIV primary cortical neurons exposed to glutamate excitotoxicity (300 μM; 5 min), incubated for 24 h and immunostained for p65 NF-κB subunit (red) and HIF-1α (green). Nuclear counterstaining is shown with DAPI. Note the nuclear re-localization of p65 indicating the activation of NF-κB signaling pathway and the increased immunostaining for HIF-1α from negative in control neurons to be detectable after excitotoxicity. Bar = 50 μm.

P-gp and EPO-R genes are known targets of transcription factors HIF-1α and NF-κB, so we tested whether the excitotoxicity was able to induce the activation of these factors. As expected, glutamate pulse induced the stabilization of HIF-1α and nuclear translocation of both p65 NF-κB subunit and HIF-1α in primary cortical neurons (Figure 3).

### rHu-EPO Inhibit the P-gp Efflux Activity

**FIGURE 4:**
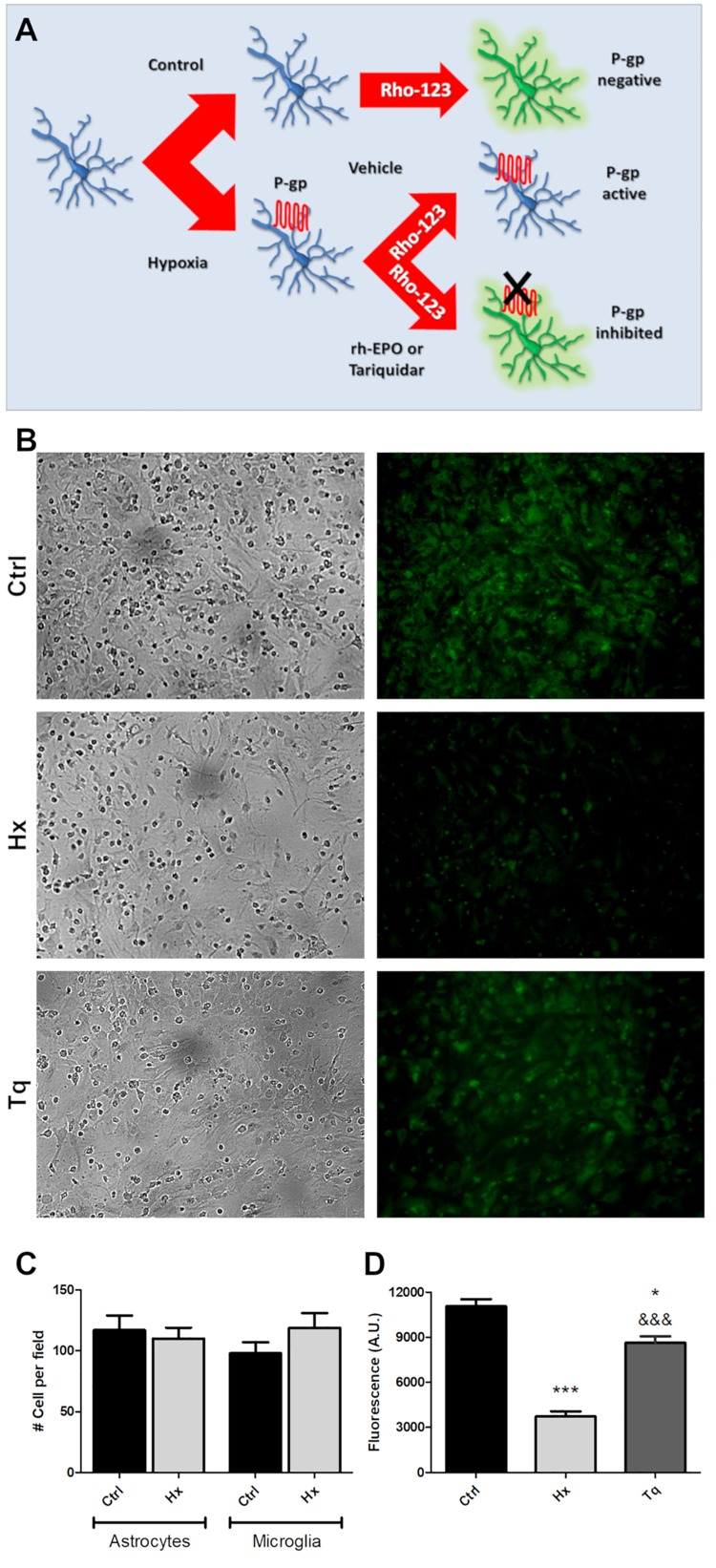
Functional assay for determine the P-gp activity after hypoxia. **(A)** Schematic procedure for induction and determination of P-gp activity through the Rhodamine 123 efflux. **(B)** Representative images of the glial cell cultured under control (Ctrl), hypoxia (Hx) or tariquidar (Tq) condition. Phase contrast images were used to check the condition of the culture and were obtained from the same field as the Rho 123 fluorescence images. **(C)** Quantitative analysis of the number of astrocytes and microglia under hypoxia. **(D)** Fluorescence quantification of the functional assay. Differences were analyzed by one-way ANOVA and Bonferroni post-test with a significance level of the *p* < 0.05 (^*^) and *p* < 0.001 (^∗∗∗^) vs. the control or *p* < 0.001 (&&&) vs. the hypoxia condition.

We have hypothesized that rHu-EPO treatment may have an effect over the P-gp activity. As a first step to test the rHu-EPO effects, we designed a functional assay using a glial mixed culture containing astrocytes and microglia. Both cell types are easily discriminated by analyzing morphological clues. Astrocytes are classically seen as star-like shaped cells growing as a monolayer on the bottom of the tissue culture plate, while microgliocytes are small rounded-shaped cells usually growing on top of astrocytic monolayer. Astroglial Rho123 retention was evaluated after chemically CoCl_2_-induced hypoxia, and P-gp specific 3rd generation P-gp blocker Tariquidar was used as positive control of P-gp inhibition. Rho123 is a cell permeable dye that is also P-gp substrate and thus Rho123-loaded cells rapidly pump out Rho123 if the P-gp is active. As shown in Figure 4A, decreased Rho123 retention level in hypoxic astrocytes was observed, while the treatment of these cells with 5 M P-gp inhibitor Tariquidar prevented the Rho123 effluxμ (Figures 4B,D). Interestingly, Rho123 retention in microglial cells remained unchanged with all treatments (Figure 4B) due to the absence of Pgp expression in this cell type. In our conditions chemical hypoxia by CoCl_2_ did not produce significant alterations in astrocyte or microglia cell survival (Figure 4C).

**FIGURE 5:**
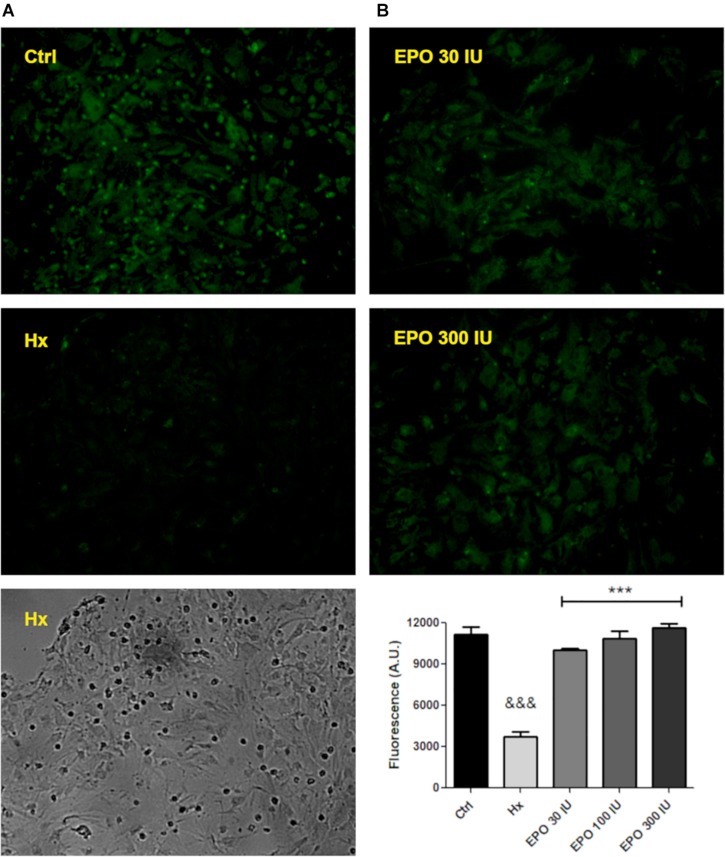
hr-EPO blocks the activity of the P-gp. **(A)** Images of Rhodamine 123 retention with different treatments (primary magnification x20). **(B)** Quantification of the rHu-EPO effect. Differences were analyzed by ANOVA with a significance level of *p* < 0.001 (^∗∗∗^compared to control and ^&⁣&&^compared to hypoxia treatment).

Next, using the same functional assay, we tested the effect of three rHu-EPO concentrations (30, 100, 300 IU/ml) on P-gp efflux activity. Again, hypoxic astrocytes showed a decreased Rho123 retention that was prevented by rHu-EPO treatment, in a concentration independent manner (Figure 5).

## Discussion

In the present study, we reported the simultaneous induction of P-gp and EPO-R expression in brain cortex after SE-induced stress. A similar induction was also observed *in vitro* when primary cultured neurons where stimulated with an excitotoxic glutamate pulse, and it was associated with NF-κB and HIF-1α nuclear translocation. These translocations are typically associated with the activation of both hypoxic and inflammatory responses. Since *in vitro* testing did not involve oxygen deprivation, the NF-κB/HIF-1α crosstalk is the most likely explanation for this cross-stimulation. Specifically, in innate immunity, this crosstalk has been already demonstrated [Rius et al., 2008; revised in D’Ignazio et al. (2016)].

Interestingly, as previously observed, other situations induce simultaneous EPO-R and P-gp expression. We have previously shown CoCl_2_ exposure, a chemical compound that inhibit prolyl-hydroxylase and thus stabilizes HIF-1α, induce both P-gp and EPO-R expression (Merelli et al., 2011). This evidence, together with our present results, suggests that severe convulsive stress can induce the expression of a wide spectrum of HIF-1α and NF-κB-dependent genes, mimicking hypoxic or inflammatory conditions (Semenza, 2000; Ruscher et al., 2002).

### Canonical and Non-canonical Response to EPO

**FIGURE 6:**
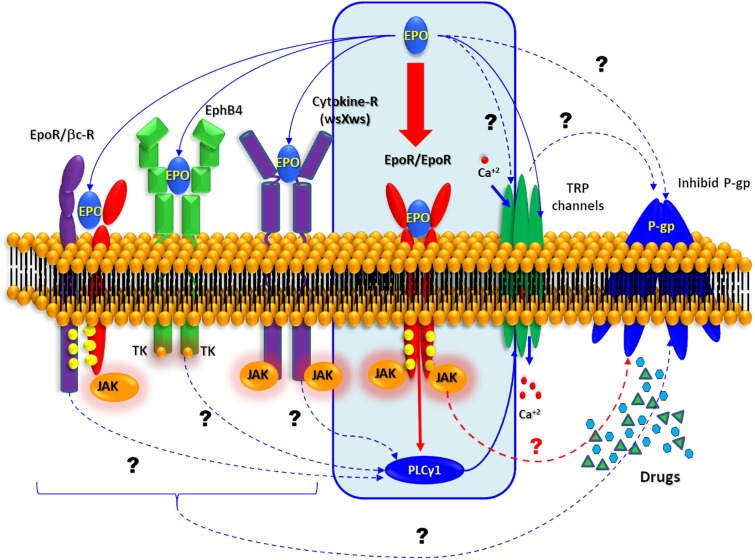
Possible EPO signaling pathway. Schematic representation of the all possible molecular mechanism. Interaction described by bibliography are represented by solid arrows while other possible target of EPO is represented by dashed arrows.

The high EPO-R expression induced by these convulsive stress signals provides an opportunity for the pharmacological intervention with EPO to seek cell protection. In this regard, it was demonstrated that EPO can trigger a wide spectrum of protective effects, including improvement in learning and memory, promoting CNS development or blocking cell death in stroke models. EPO-induced intracellular signaling is mainly mediated by the homodimerization of EPO-R (Figure 6) followed by the activation of Janus kinase 2 (JAK2) and signal transducer and activator of transcription 5 (STAT5), as well as mitogen- activated protein kinase (MAPK) and NF-κB (Maiese et al., 2004; Broxmeyer, 2013). Notably our study illustrates that glutamic acid-stimulated neurons exhibit NF-κB expression. The NF-κB-seems to mediate EPO effects depending on the type of cells involved (Nairz et al., 2012). While EPO activates NF-κB in erythroid cells, it inhibits this pathway in macrophages, resulting in decreased production of TNF-alpha and reduced expression of nitric oxide synthase.

In CNS several EPO non-canonical pathway have been summarized by Ostrowski and Heinrich (2018). EPO can activate the JAK pathway through activation of the so called common beta receptor (βcR) (Brines and Cerami, 2006; Zhang et al., 2014), the orphan cytokine receptor-like factor 3 (CRLF3) is a type 1 cytokin receptor as well as thrombopoietin receptor, prolactin receptor and EPO-R (Boulay et al., 2003). The first evidence of CRLF3 is involved in neuroprotective EPO effect proceed from the experiments with primary cultures insect neurons. Hahn et al. (2017) showed that EPO neuroprotective effect against the hypoxia was loose when they silencing the orthologous CRFL3. Additionally, experiments performed in neurons of different insects that do not express EPO-R (*Locusta migratoria*, *Chorthippus biguttulus*, and *Tribolium castaneum*) revealed Epo-mediated neuroprotection throughout JAK/STAT activation (Heinrich et al., 2017).

Working with ovarian carcinoma cells, Pradeep et al. (2015), shown that EPO was recognized by Ephrin receptor B4 (EphB4) which is well known through its interaction with Ephrin B2 (EphB2). Furthermore, EphB2 also actives the EPO-R in the same cells. In contrast to the canonical pathway (EPO/EPO-R induced-JAK2/STAT5 activation), the binding of EPO to EphB4 promotes the activation of Src kinase and STAT3. In CNS, EphB4 is expressed in neurons, astrocytes, and endothelial cells, but have “no-JAK” tyrosine kinase activity involved (Ashton et al., 2012; Todd et al., 2017) and its stimulation with EphB2 has been demonstrated to regulate the neurogenesis in the hippocampus. This effect is superimposed with EPO effect, so the stimulation of EphB4 could explain, at least in part, the neuroprotective action of EPO.

On the other hand, a new activity of EPO has been recently reported on several transient receptor potential channels (TRPs) (Myssina et al., 2003; Danielczok et al., 2017). Some of these TRPs are related to higher seizure susceptibility (Zheng and Phelan, 2014; Phelan et al., 2017). Recently it was proposed that an anticonvulsant action of TRPV1 antagonists could be mediated through the modulation of glutaminergic systems (Suemaru et al., 2018) or its relation with pentylenetetrazole and amygdala-induced kindling models (Shirazi et al., 2014). Additionally, others report, have linked some TRPs with or P-gp activity (Ma et al., 2012, 2014; Wang et al., 2015).

### Perspectives for rHu-EPO Treatment in Refractory Epilepsies

In spite the growing availability of new antiepileptic drugs, pharmacoresistant phenotype remains observed in 30–40% of patients with epilepsy. In this subset of patients, brain overexpression of P-glycoprotein and its efflux activity have been proposed as a main mechanism of refractoriness in epilepsies (Robey et al., 2008; Feldmann and Koepp, 2016; Tang et al., 2017).

In our hands, EPO effectively reduced the Rho123 efflux from astrocytes showing an effect that mimic the reached by the specific P-gp blocker Tariquidar. To this end, we currently cannot define if EPO is acting directly on P-glycoprotein or indirectly via the canonical or non-canonical EPO-R. We cannot rule out either that EPO acts independently of receptor interaction (see for revision Bond and Rex, 2014; Malik and Di Benedetto, 2018). In addition, it should be considered that our cultures contain a 20–30% microglia that can be interacting in *trans* with astrocytes to mediate EPO effects on P-gp. Seizure burden induces high expression of P-gp, and hypoxic-insult increased neuronal expression of EPO-R and P-gp, respectively (Merelli et al., 2011; Auzmendi et al., 2013). In addition, our present results show that SE robustly induces both EPO-R and P-gp in neurons; and that rHu-EPO effectively blocks P-gp-dependent transport in astrocytes. Under these conditions, rHu-EPO emerges as a tempting molecule to provide a potential opportunity to reverse the P-gp-dependent pharmacoresistant phenotype observed in high percentage of refractory epileptic cases and other pathological states.

To define whether EPO treatment can be used in refractory epilepsies, experiments with animal models of pharmacoresistant epilepsy should be performed. In this way, we should investigate the EPO administration via to achieve the inhibition of P-gp and the relationship with the most commonly used anticonvulsant drugs.

## Ethics Statement

All procedures involving animals and their care were conducted in accordance with our institutional guidelines, which comply with the NIH guidelines for the Care and Use of Laboratory Animals and the principles presented in the Guidelines for the Use of Animals in Neuroscience Research by the Society for Neuroscience, and were approved by the CICUAL committee of the School of Pharmacy and Biochemistry, University of Buenos Aires (Res. Nr. 1278/2012). All efforts were made to minimize animal suffering and to reduce the number of animals used.

## Author Contributions

AR, AL, and JA designed the experiments. AR provided supplies and equipments. All authors analyzed the results and redacted the manuscript. AM revised the text. JA developed the *in vivo* and *in vitro* experiments.

## Conflict of Interest Statement

The authors declare that the research was conducted in the absence of any commercial or financial relationships that could be construed as a potential conflict of interest.
